# Clinical Guidelines on Long-Term Pharmacotherapy for Bipolar Disorder in Children and Adolescents

**DOI:** 10.3390/jcm3010135

**Published:** 2014-01-21

**Authors:** Joanna H. Cox, Stefano Seri, Andrea E. Cavanna

**Affiliations:** 1 Department of Neuropsychiatry, University of Birmingham and BSMHFT, Birmingham B152FG, UK; E-Mail: jhc948@bham.ac.uk; 2 School of Life and Health Sciences, Aston University, Birmingham B47ET, UK; E-Mail: s.seri@aston.ac.uk; 3 Sobell Department of Motor Neuroscience and Movement Disorders, Institute of Neurology and UCL, London WC1N3BG, UK

**Keywords:** adolescents, bipolar disorder, children, guidelines, pharmacotherapy

## Abstract

Bipolar disorder is a severe affective disorder which can present in adolescence, or sometimes earlier, and often requires a pharmacotherapeutic approach. The phenomenology of bipolar disorder in children and adolescents appears to differ from that of adult patients, prompting the need for specific pharmacotherapy guidelines for long-term management in this patient population. Current treatment guidelines were mainly developed based on evidence from studies in adult patients, highlighting the requirement for further research into the pharmacotherapy of children and adolescents with bipolar disorder. This review compares and critically analyzes the available guidelines, discussing the recommended medication classes, their mechanisms of action, side effect profiles and evidence base.

## 1. Introduction

Bipolar disorder (BD), previously known as manic depression, is a complex psychiatric illness characterized by alternating episodes of mania and depression. Manic symptoms commonly include racing thoughts, delusions of self-grandeur, partaking in risky behaviors and reduced need for sleep. In contrast, during depressive periods, patients commonly experience reduced mood, changes in appetite, irritability and anhedonia. BD is considered to be one of the most disabling psychiatric disorders, associated with a suicide rate approximately 20–30 times that of the general population [1]. Overall lifetime prevalence is thought to be around 4% [2], with typical onset occurring during late adolescence or early adulthood. Prevalence of BD in children and adolescents alone is thought to be around 2% [3,4]; however there is controversy surrounding the diagnosis of BD in this patient population. Although there is no clear consensus as to how many types of BD exist even in adult patients, current classification systems acknowledge the existence of two BD subtypes [5]. BD type I is characterized by the presence of one or more manic episodes, whilst depressive or hypomanic episodes occur frequently but are not required for diagnosis. In BD type II there are no manic episodes, but one or more hypomanic episodes and one or more major depressive episodes. Hypomanic episodes do not go to the full extremes of mania (*i.e.*, do not usually cause severe social or occupational impairment, and are not associated with psychotic features).

The first controversial issue is about diagnostic criteria, as not all professionals agree on the usefulness of the criteria set out in the Diagnostic and Statistical Manual of Mental Disorders (DSM) [5]. Specifically, it has been suggested that symptoms of mania presenting in childhood are not easily distinguishable from those of attention deficit hyperactivity disorder (ADHD) and other neuropsychiatric disorders [6]. With regard to BD subtypes, there is reasonable agreement surrounding the diagnosis of BD type I in children and adolescents, however there is doubt concerning the validity of a broad diagnosis of bipolar spectrum disorder. In the United Kingdom (UK), the National Institute for Health and Care Excellence (NICE) [7] suggested that a broader bipolar diagnosis could be unreliable and therefore not useful. This is somewhat in contrast with the prevalent view in the United States, where other BD diagnoses, such as BD type II and BD-not otherwise specified, are widely accepted and in recent years there has seen a 40-fold increase [8]. The exact reason for this phenomenon is yet unknown; possible explanations include the wider application of the diagnostic criteria to children and adolescents, and previous under-diagnosis of the condition [9]. In the absence of accepted diagnostic criteria specific to children and adolescents, in pediatric practice BD is generally diagnosed based on adult criteria. In DSM-5, bipolar and related disorders are given a chapter on their own (between depressive disorders and schizophrenia spectrum disorders), where bipolar-like phenomena that do not fulfill the diagnostic criteria for BD type I, II or cyclothymic disorder (*i.e.*, short-duration hypomanic episodes and major depressive episodes, hypomanic episodes with insufficient symptoms and major depressive episodes, hypomanic episode without prior major depressive episode, and short-duration cyclothymia) are summarized under the label “other specified bipolar and related disorders”. In this new edition of the DSM, the symptoms which have to be present to fulfill the formal criteria for a hypomanic or manic episode have been specified: While in the past only a distinct period of abnormally and persistently elevated, expansive or irritable mood was necessary, these symptoms now have to be present in association with persistently increased (goal-directed) activity or energy [10].

Early-onset BD tends to be clinically more severe than later-onset forms and patients tend to have more frequent episodes [11]. Furthermore, mood swings and episodes of mixed mania and depression appear to be more frequent in younger patients [12]. Importantly, risk of suicide is also higher in early-onset BD [11]. Differences in BD phenomenology between children/adolescents and adult patients should therefore reflect in separate management guidelines for these two patient groups. Management of BC includes integrated behavioral and pharmacological treatment. The most commonly used behavioral therapies include targeted psychotherapy, family therapy, interpersonal and social rhythm therapy and group co-education, which have been shown to be effective, especially as an adjunct to pharmacological therapy [7,8]. This review will focus specifically on currently available guidelines for long-term pharmacological therapy of BD in children and adolescents.

## 2. Pharmacological Therapies for BD in Children and Adolescent: Overview

There are four main classes of medications indicated for the management of BD in children and adolescents. These include mood stabilizers (primarily lithium), atypical antipsychotics, anticonvulsant drugs and antidepressants. The mechanisms of action along with guidelines for their use in children and adolescents will be reviewed separately for each class of medication. The treatment guidelines which will be discussed here include those published by the National Institute for Health and Care Excellence (NICE) [7], the American Academy for Child and Adolescent Psychiatry (AACAP) [3], and the Child and Adolescent Bipolar Foundation (CABF) [14] (Table 1). A further set of guidelines for the diagnosis and treatment of BD in adults have been compiled by the British Association of Psychopharmacology (BAP) [8]. However, although these guidelines contain information about the diagnosis of BD in children and adolescents, they do not make any specific recommendations regarding treatment of the disorder in this patient population, and therefore will not be discussed in this review.

**Table 1:** Summary of current clinical guidelines for the pharmacological treatment of bipolar disorder in children and adolescents.

Guidelines	Year	First-line agents	Second-line agents
CABF	2005	Lithium/valproate/carbamazepine/olanzapine/quetiapine/risperidone	Alternative drug class Combination of two drug classes
NICE	2006	Atypical antipsychotic	Lithium +/− valproate
AACAP	2007	Lithium/valproate +/− atypical antipsychotics	Lamotrigine/olanzapine

CABF, Child and Adolescent Bipolar Foundation; NICE, National Institute for Health and Care Excellence; AACAP, American Academy for Child and Adolescent Psychiatry.

### 2.1. Medication Classes

#### 2.1.1. Lithium

Among the most commonly prescribed pharmacological treatments for BD are lithium salts, most commonly lithium carbonate. A number of lithium salts are used medically as mood stabilizing drugs, and are commonly referred to simply as “lithium”. The mechanism of action of lithium is complex, and multi-faceted, as it acts by mimicking the role of various cations, entering cells and interfering with transmitter release, thereby inhibiting of a number of enzymes within signal transduction pathways. This is thought to decrease neuronal over-excitability, thus reducing the symptoms of mania [15]. Lithium has been used in the treatment of BD since the 1960s, and a large number of studies have demonstrated its superiority to placebo in long-term treatment of BD [16,17], in the prevention of relapses [17] and of acute manic episodes [18]. Lithium is currently the only drug used in the treatment of BD licensed in both the UK and USA for patients with a diagnosis of BD above 12 years of age. A limited number of double-blind randomized controlled trials have been carried out to assess the efficacy of lithium as a treatment for BD in children and adolescents. Most of these studies are however limited by small sample sizes and variability in diagnostic criteria. Despite these limitations, lithium remains the most widely investigated treatment for BD in this age group. Clinical research has shown that lithium has a number of significant adverse effects, for which patients should be monitored closely. These include tremor, weight gain and dehydration. It also has a relatively narrow therapeutic index and therefore precise dosing is required, alongside monitoring of blood lithium levels. NICE recommends that lithium should be used as a second-line agent in the long-term treatment of children and adolescents, whereas AACAP and the CABF guidelines suggest lithium as one of the possible first-line agents.

#### 2.1.2. Atypical Antipsychotics

Second-generation or atypical antipsychotics, especially olanzapine, quetiapine and risperidone, have recently been advocated for the treatment of BD [19]. Atypical antipsychotics mainly differ from their first-generation counterparts in their improved safety profile. Side effects of atypical antipsychotics include tardive dyskinesia and extrapyramidal symptoms (although to a lower extent than first generation antipsychotics), weight gain and metabolic changes. The exact mechanisms of action of atypical antipsychotics are not fully understood and vary between individual drugs. All antipsychotics have an inhibitory action on the dopaminergic neurotransmitter system; however the receptors involved, and the affinity to which they bind, differs between agents [20]. Few studies have specifically evaluated the efficacy of atypical antipsychotics in the treatment of BD in children and adolescents, and the recommendations published in the guidelines are primarily extrapolated from adult patients’ data. Aripiprazole, olanzapine, risperidone and quetiapine have been shown to be effective in the management of acute mania in adults, whilst olanzapine has also been proven useful in the maintenance therapy for BD [13]. The NICE guidelines suggest that due to their superior safety profile compared to lithium, atypical antipsychotics should be the first-line treatment for the long-term maintenance of BD in children and adolescents. CABF recommendations suggest that atypical antipsychotics may be used as a first-line treatment, as an alternative to lithium or anticonvulsants. No preference is suggested for any specific drug, as the comparative efficacy of the drugs had not been sufficiently investigated. Finally, the AACAP guidelines suggest that atypical antipsychotics, without specifying individual agents, may be used as an adjunct or alternative to pharmacotherapy with lithium and sodium valproate.

#### 2.1.3. Anticonvulsant Drugs

Anticonvulsants are medications originally developed to prevent and treat epileptic seizures. They are however increasingly being used in conditions other than epilepsy, such as neuropathic pain and BD, due to their mood stabilizing properties [21]. Common side effects of anticonvulsants include drowsiness, weight gain, dizziness and diplopia. Sodium valproate also poses considerable caution due to its teratogenic potential. These medications appear to be particularly effective for the treatment of acute manic episodes and prevention of further episodes. Anticonvulsant drugs most commonly recommended in clinical guidelines concerning the treatment of BD in children and adolescents include sodium valproate, carbamazepine and lamotrigine. Sodium valproate with its GABAergic mechanism of action [22], is the most frequently recommended anticonvulsant in the treatment of BD. In the NICE guidelines, sodium valproate is considered as a second line agent (in patients who do not respond to atypical antipsychotic monotherapy) as an alternative to lithium, in male patients. Lithium alone is recommended as a second-line treatment in female patients due to the teratogenic effects of sodium valproate. The AACAP guidelines provide different recommendations, suggesting that valproate may be used as a first-line agent alongside lithium, with or without antipsychotics. The use of lamotrigine, a sodium channel blocker with known antidepressant action, is also mentioned as an effective maintenance therapy for children and adolescents with BD. The guidelines published by the CABF also recommend sodium valproate as a second or third line treatment, which may be complemented by other anticonvulsants such as carbamazepine (a stabilizer of voltage-gated sodium channels).

#### 2.1.4. Antidepressant Drugs

The majority of the drugs mentioned above are effective in the treatment of mania, however depression is also an issue of primary importance in BD. Antidepressant drugs, most commonly selective serotonin reuptake inhibitors (SSRIs) are also commonly prescribed in patients with BD. These antidepressants work by preventing reuptake of serotonin back into the presynaptic membrane, therefore increasing the amount of serotonin available to be taken up by the postsynaptic membrane [23]. SSRIs are relatively safe medications; however occasional side effects can include headache, nausea, agitation and sexual dysfunction. In the NICE guidelines, it is recommended that in children and adolescents with bipolar depression, initial treatment should consist of their normal mood stabilizer alongside psychotherapy. If after four weeks the response is minimal, SSRI treatment is recommended. Fluoxetine is recommended in the first instance, and if this produces no response, alternatives such as sertraline or citalopram may be tried. The AACAP guidelines recommend SSRIs as a useful adjunct when the patient is taking at least one mood stabilizer. Their published article also mentions the demonstrated efficacy of the combination of olanzapine, an atypical antipsychotic, and fluoxetine in treating bipolar depression in adults with BD, suggesting that this may also be a worthwhile combination in treatment of children and adolescents. The CABF produced no algorithm for pharmacotherapy of bipolar depression, as they did for mania, due to insufficient evidence pertaining to antidepressant treatment in this patient group. They did however, mention the potential benefits of SSRIs or bupropion, an atypical antidepressant thought to stimulate release and prevent reuptake of both dopamine and noradrenaline.

### 2.2. Guidelines

#### 2.2.1. National Institute for Health Care and Excellence (NICE) Guidelines

Clinical guidelines produced by NICE consist of recommendations on treatment of specific conditions within the NHS in the United Kingdom. They also publish guidance pertaining to public health and interventional procedures, alongside appraisals of medical technology. Their guidance is intended for use not only by health care professionals, but also by local authorities, employers or any other professional body. NICE Clinical Guideline 38 was published in 2006 and covers management strategies for patients with BD, with focus on treatment of BD in adults, children and adolescents in primary and secondary care. These guidelines are based on high quality studies, reflecting the best available evidence, and include recommendations on both pharmacotherapy and psychological therapy, as well as advice on self-help and the support available for family and carers.

#### 2.2.2. American Academy of Child and Adolescent Psychiatry (AACAP) Guidelines

The “Practice Parameter for the Assessment and Treatment of Children and Adolescents with Bipolar Disorder” was developed by the AACAP, and published in the Journal of the American Academy for Child and Adolescent Psychiatry in 2007. The focus of these guidelines was to encourage best practices in child mental health, and consist of a number of recommendations pertaining to the diagnosis and management of children and adolescents with BD. They are based mainly on studies assessing treatments of BD in adult patients, and suggest that treatment of children and adolescents should consist of a drug approved by the Food and Drug Administration (FDA) for use in adults. They suggest that the chosen drug should be selected based on six factors, including evidence of efficacy, phase of the illness, presence of confounding presentation, drug’s side-effect profile, patient’s medication history and personal/family preference.

#### 2.2.3. Child and Adolescent Bipolar Foundation (CABF) Guidelines

The CABF, a child psychiatric workgroup, developed a set of guidelines consisting of two treatment algorithms regarding BD in children and adolescents. These algorithms suggest treatment pathways for manic episodes, with or without psychosis, in children and adolescents with BD. They were compiled by CABF members along with 20 clinicians and were published in the Journal of the American Academy for Child and Adolescent Psychiatry in 2005. These treatment guidelines are very concise, specific and easy to follow. Furthermore, the authors have recognized the paucity of large-scale studies involving this patient population, and did not produce an algorithm for depression in BD. However, the algorithms they did produce were based primarily on studies involving children and adolescents, and not solely adult studies.

## 3. Comparison and Critique of Guidelines

These three sets of guidelines were published by three different organizations, and have a slightly different remit. Both the AACAP and the CABF guidelines were developed specifically for the treatment of children and adolescents with BD, whereas the NICE clinical guidelines are primarily focused on treatment of adults with BD, with a short section pertaining to children and adolescents. The differences and similarities between each set of guidelines deserve a few comments, along with a discussion of the discrepancies. Lithium is the most frequently prescribed medication for adult BD, however there are differences concerning its use in children and adolescents across these guidelines: AACAP and CABF recommend lithium as a first-line agent in the long-term treatment of BD in children and adolescents, whereas NICE recommends an atypical antipsychotic with fewer adverse effects such as weight gain and elevated prolactin. This seems to suggest that the NICE guidelines prioritizes patient safety and compliance (reduced undesired effects), in addition to the evidence base for treatment. Furthermore, the NICE guidelines have stipulated that sodium valproate is not recommended for treatment of female patients due to its teratogenic effects, whereas neither the AACAP nor the CABF made any distinction between management of male and female patients. Apart from a few differences, these guidelines are on the whole consistent, recommending the same drug classes, and where specific drugs are indicated, these are generally the same. As the guidelines were all produced at around similar times, it is likely that they are based on similar, or largely overlapping, research studies. Importantly, all three guidelines recognize the lack of evidence specific to children and adolescents, and base their clinical recommendations on evidenced derived from adult studies. There is a clear need for data from more double-blind, randomized controlled trials in order to update evidence-based recommendations. Overall, these guidelines call for greater attention from both clinicians and researchers in the field towards the early diagnosis of BD in childhood and adolescence and the consideration of early intervention and long term follow up as required, based on the subtle aspects of individual presentation of the condition.

## 4. Conclusions

At present, very few double-blind randomized controlled trials have been carried out in child and adolescent patients with BD. Available guidelines have been largely developed based on studies carried out on adult samples. It is generally accepted that clinical features of BD in children and adolescents varies significantly from those of adult-onset, confirming the need for more trials specifically in the younger age-groups. Inevitably, this raises ethical issues as children and adolescents with BD can be a vulnerable patient group, both due to their psychiatric condition and their young age, and may lack the capacity to appropriately consent to participation in research. The choice of management strategies is also complicated by the fact that affective disorders, including BD, are a common psychiatric co-morbidity in patients with underlying neurological disorders [24]. Finally, all three sets of guidelines analyzed in this review were published more than five years ago, highlighting the need for updated evidence-based recommendations.

## References

[1] Pompili M., Gonda X., Serafini G., Innamorati M., Sher L., Amore M., Rihmer Z., Girardi P. (2012). Epidemiology of suicide in bipolar disorders: A systematic review of the literature. Bipolar Disord..

[2] Kessler R., Berglund P., Demler O., Jin R., Merikangas K.R., Walters E.E. (2005). Lifetime prevalence and age-of-onset distributions of DSM-IV disorders in the National Comorbidity Survey Replication. Arch. Gen. Psychiatry.

[3] McClellan J., Kowatch R., Finding R. (2007). Practice parameter for the assessment and treatment of children and adolescents with bipolar disorder. J. Am. Acad. Child Adolesc. Psychiatry.

[4] Van Meter A., Moreira A., Youngstrom E. (2011). Meta-analysis of epidemiologic studies of pediatric bipolar disorder. J. Clin. Psychiatry.

[5] American Psychiatric Association (2013). Diagnostic and Statistical Manual of Mental Disorders.

[6] Biederman J. (1998). Resolved: Mania is mistaken for ADHD in prepubertal children. J. Am. Acad. Child Adolesc. Psychiatry.

[7] National Institute for Health and Care Excellence (2006). Bipolar Disorder—The Management of Bipolar Disorder in Adults, Children and Adolescents in Primary and Secondary Care (CG38).

[8] Goodwin G. (2009). Evidence-based guidelines for treating bipolar disorder: Revised second edition—Recommendations from the British Association for Psychopharmacology. J. Psychopharmacol..

[9] Leibenluft E., Rich B. (2008). Pediatric bipolar disorder. Annu. Rev. Clin. Psychol..

[10] Angst J. (2013). Bipolar disorders in DSM-5: Strengths, problems, perspectives. Int. J. Bipolar Disord..

[11] Perlis R., Miyahara S., Marangell L., Wisniewski S.R., Ostacher M., DelBello M.P., Bowden C.L., Sachs G.S., Nierenberg A.A., STEP-BD Investigators (2004). Long-term implications of early onset in bipolar disorder: Data from the first 1000 participants in the systematic treatment enhancement program for bipolar disorder. Biol. Psychiatry.

[12] Birmaher B., Axelson D., Strober M., Gill M.K., Valeri S., Chiappetta L., Ryan N., Leonard H., Hunt J., Iyengar S., Keller M. (2006). Clinical course of children and adolescents with bipolar spectrum disorders. Arch. Gen. Psychiatry.

[13] Derry S., Moore R.A. (2007). Atypical antipsychotics in bipolar disorder: Systematic review of randomised trials. BMC Psychiatry.

[14] Kowatch R., Fristad M., Birmaher B., Wagner K.D., Findling R.L., Hellander M. (2005). Child Psychiatric Workgroup on Bipolar Disorder. Treatment guidelines for children and adolescents with bipolar disorder. J. Am. Acad. Child Adolesc. Psychiatry.

[15] Jope R. (1999). Anti-bipolar therapy: Mechanism of action of lithium. Mol. Psychiatry.

[16] Fawcett J., Kravitz H. (1985). The long-term management of bipolar disorders with lithium, carbamazepine, and antidepressants. J. Clin. Psychiatry.

[17] Geddes J., Burgess S., Hawton K., Jamison K., Goodwin G.M. (2004). Long-term lithium therapy for bipolar disorder: Systematic review and meta-analysis of randomized controlled trials. Am. J. Psychiatry.

[18] Poolsup N., Po L., de Oliveira I. (2000). Systematic overview of lithium treatment in acute mania. J. Clin. Pharm. Ther..

[19] Yatham L., Goldstein J., Vieta E., Bowden C.L., Grunze H., Post R.M., Suppes T., Calabrese J.R. (2005). Atypical antipsychotics in bipolar depression: Potential mechanisms of action. J. Clin. Psychiatry.

[20] Seeman P. (2002). Atypical antipsychotics: Mechanism of action. Can. J. Psychiatry.

[21] Macdonald K., Young L. (2002). Newer antiepileptic drugs in bipolar disorder. CNS Drugs.

[22] Czapinski P., Blaszczyk B., Czuczwar S. (2005). Mechanisms of action of antiepileptic drugs. Curr. Top. Med. Chem..

[23] Feighner J. (1999). Mechanism of action of antidepressant medications. J. Clin. Psychiatry.

[24] Jones R., Rickards H., Cavanna A.E. (2010). The prevalence of psychiatric disorders in epilepsy: A critical review of the evidence. Funct. Neurol..

